# Use of Two-Photon Lithography with a Negative Resist and Processing to Realise Cylindrical Magnetic Nanowires

**DOI:** 10.3390/nano10030429

**Published:** 2020-02-28

**Authors:** Joseph Askey, Matthew Oliver Hunt, Wolfgang Langbein, Sam Ladak

**Affiliations:** School of Physics and Astronomy, Cardiff University, The Parade, Cardiff CF24 3AA, UK; askeyjw@cardiff.ac.uk (J.A.); huntmo@cardiff.ac.uk (M.O.H.); langbeinww@cardiff.ac.uk (W.L.)

**Keywords:** two-photon lithography, Bloch point, magnetic nanowire, 3D nanomagnetism, electrodeposition, micro-magnetic simulations, magnetic force microscopy

## Abstract

Cylindrical magnetic nanowires have been shown to exhibit a vast array of fascinating spin textures, including chiral domains, skyrmion tubes, and topologically protected domain walls that harbor Bloch points. Here, we present a novel methodology that utilizes two-photon lithography in order to realize tailored three-dimensional (3D) porous templates upon prefabricated electrodes. Electrochemical deposition is used to fill these porous templates, and reactive ion etching is used to free the encased magnetic nanowires. The nanowires are found to have a diameter of 420 nm, length of 2.82 μm, and surface roughness of 7.6 nm. Magnetic force microscopy in an externally applied field suggests a complex spiraling magnetization state, which demagnetizes via the production of vortices of alternating chirality. Detailed micro-magnetic simulations confirm such a state and a qualitative agreement is found with respect to the switching of experimental nanowires. Surprisingly, simulations also indicate the presence of a Bloch point as a metastable state during the switching process. Our work provides a new means to realize 3D magnetic nanowires of controlled geometry and calculations suggest a further reduction in diameter to sub-200 nm will be possible, providing access to a regime of ultrafast domain wall motion.

## 1. Introduction

Three-dimensional (3D) magnetic nanowires (NW) have recently been subject to intense study due to their potential for realizing novel spin textures and ultrafast phenomena such as the spin-Cherenkov effect [1], as well as possible applications in ultrahigh density storage [2]. Such systems have typically been manufactured using electrodeposition into ion track etched [3] or anodized alumina templates [4]. For example, these methodologies have been used to realize simple cylindrical magnetic nanowires within the Bloch point domain wall regime [5], multi-segmented FeCo/Cu NWs [4], Co/Au NWs [6], and FeCoCu/Cu NW arrays [7]. Magnonic and magnetoelectric phenomena in cylindrical magnetic NWs have also been studied extensively using anodized alumina templates. Specifically, time-resolved magneto-optical Kerr effect microscopy [8] showed the presence of both uniform and quantized spin wave modes within 35 nm diameter nanowires, whilst electrical characterization has elucidated the impact of finite size effects [9], electron-phonon, and spin-wave contributions to the resistivity [10].

Two-photon lithography (TPL) is a novel fabrication methodology that has the capability of manufacturing 3D nanostructures of arbitrary geometry and by design.

The process uses a femtosecond (or picosecond) pulsed laser with wavelength typically in the infra-red, focused into a diffraction limited spot within a photoresist. The simultaneous absorption of two photons excites an electronic transition within photoinitiator molecules, yielding polymerization or de-polymerization within the photoresist. Since two-photon absorption is a third-order non-linear optical process, it only occurs within a tightly bound region within the focal volume where the intensity is sufficiently large. Translation of the focal volume with respect to the substrate then allows the production of any 3D geometry within the polymer [11].

Traditionally TPL has been used to realize photonic crystals [12], microscale optical elements [13,14], and artificial biological structures [15]. More recently, TPL has been used to realize various 3D magnetic nanostructures [16,17,18]. For example, TPL was utilized with a positive photoresist and Co electrodeposition in order to realize complex 3D tetrapod structures [17], and spin-polarized scanning electron microscopy was used to image the domain structure. The systems were found to be multi-domain, and the energy scales for magnetocrystalline anisotropy and the demagnetization field yielded spins with a distribution of angles around the nanowire long axes. These 3D tetrapod structures were also studied in time-resolved MOKE to characterize and investigate the spin-wave modes at the junctions of the adjoining wires [18]. A key issue identified within these studies was dark erosion occurring during the development stage of fabrication. If one simply considers the size of a written voxel before development, one obtains a lateral feature size of 280 nm. However, with structures written within a positive photoresist, the developer has to diffuse through the entire 3D written geometry, which takes a significant amount of time. During this time, the developer is also removing a small portion of the unexposed resist, effectively widening the pore. It is this process, known as dark erosion [19], which severely limits the prospect of single domain structures. Indeed, when one takes into account the dark erosion rate (~5 nm/min) and the rather long development times, the minimum feature size becomes approximately 430 nm [17].

The use of a negative resist is an interesting route to surpass such limitations. The minimum attainable pore diameter in a negative resist can be calculated using a modified Abbe diffraction limit [20] and typical experimental parameters (see Materials and Methods). Here, we find a minimum attainable pore diameter of 197 nm, a factor of ~2 times smaller than the positive tone example. Furthermore, dark erosion processes for negative tone photoresists are negligible, meaning such feature sizes are truly accessible. One disadvantage of this approach is the requirement to fabricate a polymeric template over the entire electrode, which necessitates the need for a reduced electrode area which can be achieved using standard UV lithography and wet chemical etching.

Here we show proof-of-principle of such a fabrication strategy that utilizes a negative-tone photoresist, which exhibits negligible dark erosion.

## 2. Materials and Methods

### 2.1. Fabrication of Ni Nanowires

**Figure 1:**
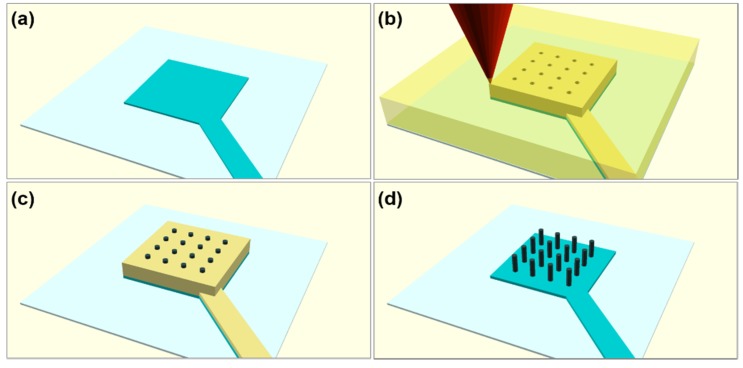
Schematic of the four steps used to realize Nickel nanowires. (**a**) The patterned indium tin oxide (ITO) electrode. (**b**) The three-dimensional (3D) porous polymer template fabricated using two-photon lithography (TPL). (**c**) Filling of the pores via electrodeposition. (**d**) Free-standing nanowires post-reactive ion etching.

Using conventional optical lithography and wet etching, a glass/indium tin oxide (ITO) 500 nm substrate was patterned into a single ITO track with a terminating square (Figure 1a). This serves as the conductive substrate upon which a porous template can be fabricated with TPL.

TPL was carried out using a commercially available system (Professional GT, Nanoscribe, Eggenstein, Germany) in the oil-immersion configuration. A negative photoresist (IP-L 780, Nanoscribe) was drop-cast onto the ITO electrode and TPL was used to fabricate the desired 3D template. Three-dimensional (3D) porous blocks were first generated using free, open source computer aided design software (OpenSCAD) with dimensions of 300 μm × 300 μm × 4 μm, as shown schematically in Figure 1b. The pores varied in diameter from 800 to 740 nm in the pattern design. The actual pore diameter after TPL was smaller than the design due to the finite lateral voxel size. Following TPL, the templates were developed in propylene glycol methyl ether acetate for 20 min in order to remove the unexposed photoresist, washed in isopropyl, and dried with compressed air.

An electrolytic Watts bath, containing nickel sulphate (225 ± 1 g/L), nickel chloride (30 ± 0.1 g/L), boric acid (30 ± 0.1 g/L), and sodium dodecyl sulfate (1 ± 0.1 g/L) heated to a temperature of 50 ± 5 °C was used to infiltrate the template pores via electrodeposition. A two-terminal setup was used where Ni blocks suspended in a Ti cage act as the anode and the patterned sample acts as the cathode, connected via a commercially available potentiostat (VersaSTAT 4000, Oak Ridge, TN, USA). A static potential of 5 ± 0.01 V was applied for 120 s, yielding wires as shown schematically in Figure 1c.

Finally, the resist template was removed by exposure to oxygen plasma (Inseto, Venus Series, Carson City, NV, USA) for 1 h at 120 W and a flow rate of 30 cm^3^/s, yielding freestanding Ni NWs (Figure 1d).

### 2.2. Physical and Magnetic Characterisation of Single Ni Nanowires

An atomic force microscope (AFM, Bruker Dimension 3100, Coventry, UK) tip was used to micro-manipulate the NWs such that their axes lay on the substrate surface. This was achieved in tapping mode by first moving the AFM tip far from the wire, carefully lowering the amplitude set-point until the tip made suitable contact with the surface, then adjusting the probe position such that the tip gradually toppled the NW. The sample was inspected under an optical microscope and then AFM and scanning electron microscopy (SEM, Hitachi Regulus 8230, Tokyo, Japan) were used to determine the dimensions and surface roughness of the nanowires.

In order to study the switching process, magnetic force microscopy (MFM, Bruker Dimension 3100, Coventry, UK) was carried out in tapping mode (lift height 80 nm) using a low moment (5 × 10^−14^ emu) tip. MFM images were obtained over the field range 0–10 mT. 

Micro-magnetic simulations were performed using the package MuMax3 [21], which solves the Landau–Liftshitz equation using a finite-difference discretization of the 3D Ni NW. The simulated NW (assumed to be a uniform cylinder) had a diameter of 420 nm, a length of 2.82 μm to emulate the physical system obtained, and a 3 nm mesh element size. The simulated material had a saturation magnetization of M_s_ = 4.9 × 10^5^ A/m, an exchange constant of A_ex_ = 9 × 10^−12^ J/m [22], and the magnetocrystalline anisotropy was assumed to be zero due to randomly oriented crystallites. MFM contrast was evaluated by computing the demagnetizing field (H_dz_) above the nanowire and taking its first derivative with respect to z at a plane of fixed height of 80 nm.

## 3. Results

### 3.1. Physical Characterisation

**Figure 2:**
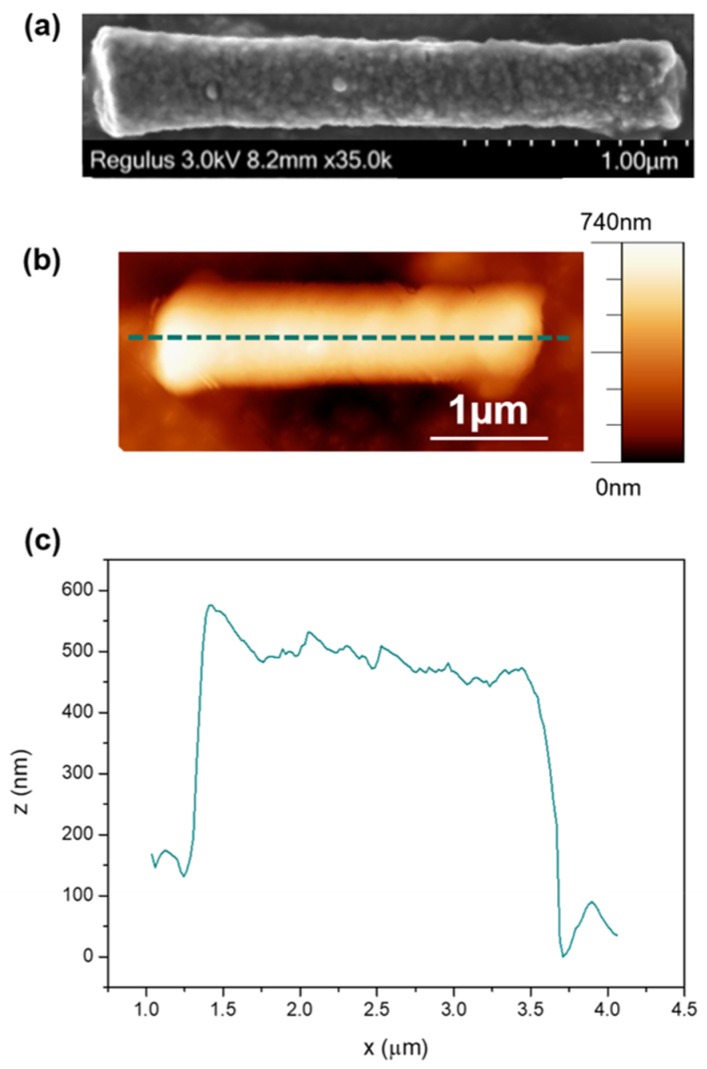
Physical characterization of a single Ni nanowire. (**a**) Scanning electron microscopy (SEM) image. (**b**) Atomic force microscope (AFM) image. (**c**) Line profile as indicated by dashed line in (**b**).

Figure 2a shows a SEM image of the nanowire which has a length of 2.82 ± 0.01 μm and a diameter that varies between 469 ± 34 and 348 ± 34 nm. Figure 2b shows an AFM image and Figure 2c shows a line profile through the center of the wire. The RMS roughness [23] of the wire was found to be 7.6 nm. Whilst the crystal structure of the Ni NW was not measured in this work, similar NWs have been reported extensively in the literature to be fcc [24,25], with a preferential growth direction along (220) [26].

### 3.2. Magnetic Characterisation

**Figure 3:**
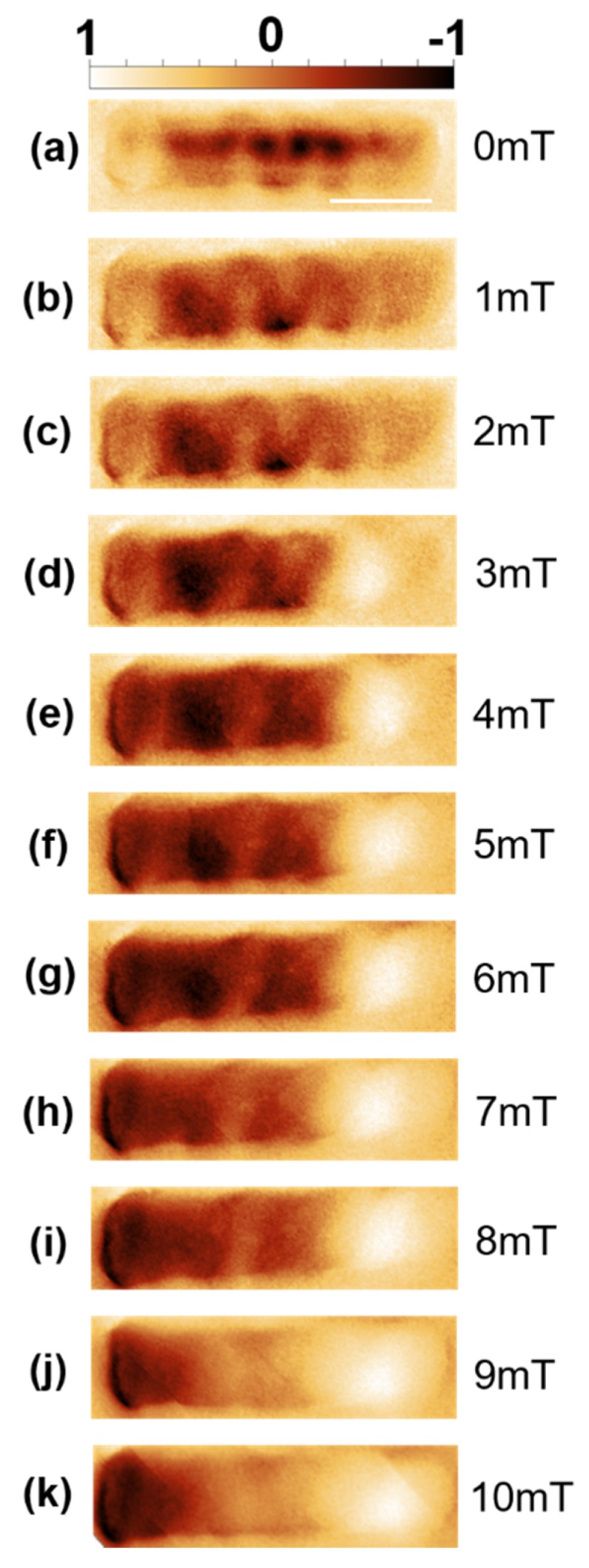
Collection of magnetic force microscopy (MFM) images taken under an externally applied field. The magnetic field varies from 0 mT, in (**a**), to 10 mT, in (**k**), in 1 mT steps (**b**–**j**). Scale bar 1 µm. Color scale normalized ±1.

Figure 3a shows the MFM image obtained at remanence after saturation in the negative field direction. The internal structure seen throughout the wire suggests a more complex state than a simple single domain. Application of 1 mT field increments to 2 mT (Figure 3b,c) shows a reduction of contrast at the wire ends and a more intricate connected internal structure consisting of oscillations across the wire length. Further field increments up to 4 mT (Figure 3d) yields contrast at the ends opposite to that seen in Figure 3a, suggesting a switched horizontal component of the magnetization. Additionally, the intricate structure within the wire has started to fade, which continues with increments up to 10 mT (Figure 3e–k), until strong contrast is only visible at the wire tips (Figure 3k).

Since MFM is sensitive to field gradients, $\frac{dH_{z}}{dz}$, due to the demagnetization field of the wire, interpretation of contrast is not straightforward for complex magnetization profiles. However, recent publications detailing magnetic reversal modes in cylindrical nanowires offer some insight. For example, MFM on demagnetized multi-segmented CoNi/Ni NWs [25] yielded structures similar to what is seen in Figure 3b,c. In this study, micromagnetic simulations suggested a vortex domain structure spanning the NW, with MFM contrast originating at points where vortex chirality switched.

**Figure 4:**
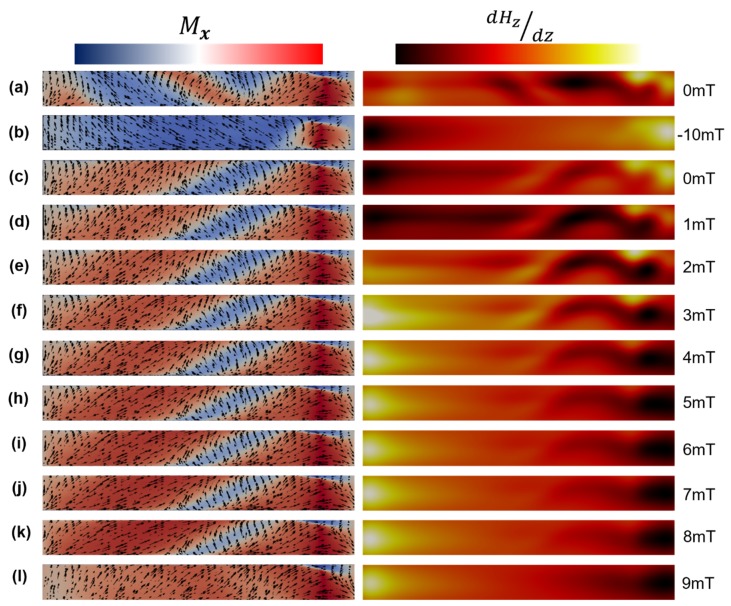
Micromagnetic simulations. Magnetization profiles (left) and corresponding calculated MFM contrast (right). (**a**) illustrates the wire at remanence with no magnetic history, (**b**) saturation at −10 mT, (**c**) resulting remnant state at 0 mT. (**d**–**l**) states after sequential field increments of 1 mT up to 9 mT. Scales for axial magnetization and MFM contrast are normalized ±1. The cylinder is 420 nm in diameter and 2.82 µm in length.

**Figure 5:**
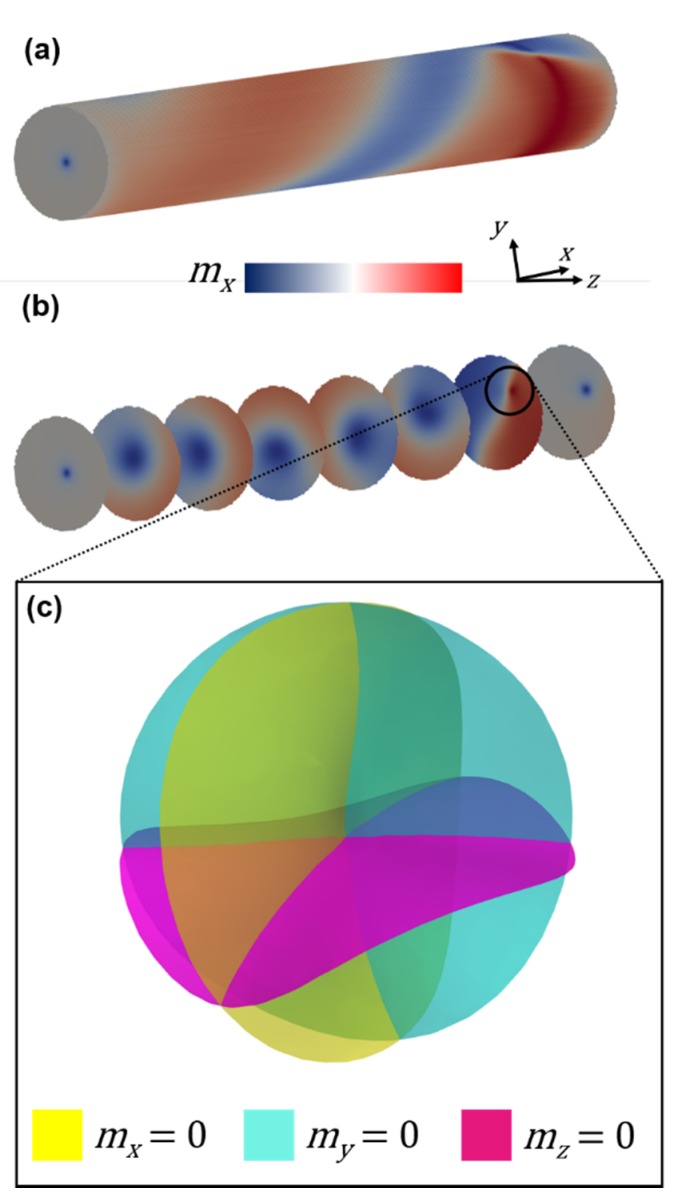
(**a**) Rotated and (**b**) cross-sectional views on the wire at 0 mT after saturation at 10 mT along long-axis (x-direction). Color scale normalized ±1. (**c**) Isosurfaces plotted in spherical region within the wire, as indicated by circled region in (**b**). Intersection of *m_x_* = *m_y_* = *m_z_* = 0 isosurfaces demonstrates the existence of the Bloch point.

Figure 4a (left) shows the simulated magnetization configuration of the outer surface of the cylinder. Here, the magnetization has been allowed to relax in absence of applied field and is seen to consist of vortex domains that spiral across the circumference of the NW. Contrary to what was seen in CoNi segmented NWs [25], the domains are found to have a component along the nanowire long axis. Figure 4a (right) shows the corresponding MFM contrast. The contrast is seen to originate from abrupt changes in magnetization, at domain boundaries, and at edges. Application of a −10 mT field leads to almost the entire NW converging into a single spiraling domain of fixed chirality (Figure 4b left) and an axial component along the field direction. The calculated MFM contrast shows opposing contrast at the edges that decays into the length of the NW (Figure 4b, right). Subsequent reduction of the field to zero leads to the creation of two spiraling vortex domains (Figure 4c), similar to Figure 4a. The application of two 1 mT field steps (Figure 4d,e) increases the canting between spins of two adjacent spiral domains, resulting in a greater MFM contrast corresponding to that boundary. A further increase in the field leads to growth of the spiral domain with a component along the external field direction at the expense of the spiral domain with a component opposite to this direction (Figure 4f–k left), leading to a fading in MFM contrast (Figure 4f–k right). At 9 mT, the NW consists mainly of a single spiral domain with a component along the external field direction (Figure 4l). The corresponding MFM contrast again is strongest at the ends and fades moving towards the center. A view of the vortex terminating at the end of the wire is shown in Figure 5a. The internal structure is illustrated in Figure 5b, where the core is seen to be narrow and confined to the edge but broadening as it moves along the axial direction and rotates counterclockwise. In the seventh cross-section in Figure 5b, a complex spin structure is seen which consists of a vortex and anti-vortex pair on opposite surfaces of the wire connected by a string of head-to-tail magnetization vectors, as shown by the white radial line. Closer inspection of the internal structure reveals the presence of a Bloch point [5], adjacent to this complex spin structure, as demonstrated by the intersection of the *m_x_* = *m_y_* = *m_z_* = 0 isosurfaces in Figure 5c. This Bloch point arises due to the convergence of two axial vortex core tubes with opposing magnetization directions, one which extends the bulk of the wire, and the other directed towards the complex spin structure mentioned above.

## 4. Discussion

The reduced NW diameter (420 nm) of the experiment when compared to the design (~740 nm) is due to the ellipsoidal point-spread function of the focused laser being centered on the trajectory of the design, and therefore, the pores are reduced by a length equivalent to the voxel diameter. The lateral TPL voxel radius can be estimated to be [27]

(1) $$r_{xy}=\frac{0.32\lambda }{\sqrt{2}{\;\mathrm{NA}}^{0.91}}$$

which for a wavelength of λ = 780 nm and a numerical aperture of NA = 1.4 yields a lateral voxel diameter of 2*r_xy_* = 264 nm. Considering both the laser tracing around the designed pores and the error in galvo positioning stage, it yields an estimated fabricated pore diameter of 476 ± 20 nm, close to our experimentally observed value. The modified two-photon Abbe formula can be written as [20]

(2) $$a_{xy}=\frac{\lambda }{2\sqrt{2}\;\mathrm{NA}}$$

which determines the resolution, defined as the distance between two distinguishable features. Using the parameters listed above, we find *a_xy_* = 197 nm, suggesting further optimization will allow access to single domain NWs and the Bloch point domain wall regime.

The NW diameter, 420 nm, is smaller than the reported diameters for the TPL fabricated Co NWs [17], 435 nm, albeit by only 15 nm. Further, the roughness of the Ni NW is a factor of ~2 times larger than that of the Co NWs. However, the reported diameter in this work is not the minimum feature size attainable. Simple improvements to the template design such as a reduction in the designed pore diameter and optimization of process parameters will yield NWs closer to the resolution limit with reduced roughness.

Though the micromagnetic simulations show some deviations to the MFM experimental results, we note several qualitative similarities. We note that there is close similarity between simulations and experiments at high fields (Figure 3k and Figure 4d). Here, the contrast seen at the edges does not end abruptly as seen in single domain systems. Instead, a slow fading in contrast is seen when moving towards the center of the NW, suggesting a gradual change in spin texture. Secondly, we note that the application of a small field leads to qualitatively similar behavior in both simulations and experiments. In both cases, the field leads to the production of oscillatory features in the MFM signal, which then fade as the field is increased, though the number of oscillatory features in the simulation is significantly lower.

A recent study, simulating FeCo modulated diameter cylindrical NWs [28], showed canted vortex domains throughout nanowires of 130 nm diameter. It was found that the system would either relax into a single vortex domain or multiple vortex domains of alternating chirality, depending on the level of disorder and the specific granular structure. For the Ni NWs studied here, having M_s_ ~4.9 × 10^5^ A/m, which is an order of magnitude lower than that of FeCo (~2 × 10^6^ A/m), larger diameters are required before the canted vortex state is achieved, but we also suggest that the grain structure and roughness play important roles in the magnetization process. For our experimental NW, the surface roughness yields magnetic surface charges which may promote the production of alternating chirality domains reducing the demagnetization energy. This surface roughness was not accounted for in our simulations.

## 5. Conclusions

In conclusion, TPL was utilized with a negative tone photoresist and electrodeposition to fabricate 3D cylindrical magnetic Ni NWs. The magnetic structure was investigated using MFM and the contrast suggests the nanowire demagnetizes via the production of vortices of opposing chirality. Micromagnetic simulations captured some of the essential features of the magnetization process and any discrepancies are likely to be due to granularity and roughness in the experimental system. Surprisingly, a Bloch point was found to be stabilized within the simulated structure during the magnetic reversal process, potentially providing another system to study these fascinating topological defects in magnetization. Real space, non-perturbative imaging modes such as photoemission electron microscopy [29] and high resolution scanning Hall microscopy [30] will be essential in such studies.

Further optimization of the TPL process will enable the fabrication of nanowires with diameters below 200 nm, allowing access to ultrafast domain walls but with the flexibility of being able to produce cylindrical geometries with defects and curvature. Such advanced lithography processes would also enable artificial spin-ice systems [31,32,33,34,35] in 3D geometries.
